# Cytomegalovirus Associated Duodenal Ulcer and Duodenitis in a Malnourished Pediatric Patient

**DOI:** 10.1155/2017/2412930

**Published:** 2017-10-08

**Authors:** Rachel Bernard, Ghanim Aljomah, Emily Klepper, Elizabeth McDonough

**Affiliations:** ^1^Pediatric Residency Program, Our Lady of the Lake Children's Hospital, Baton Rouge, LA, USA; ^2^Our Lady of the Lake Children's Hospital, Baton Rouge, LA, USA

## Abstract

Cytomegalovirus (CMV) duodenitis is a rare occurrence, especially in pediatric patients. A thirteen-month-old female presented to the Emergency Department for a febrile seizure. She was incidentally admitted for severe malnutrition with an initial workup remarkable for only a slight elevation in her ALT at 48. The patient was found to have an oral aversion requiring nasogastric tube feeds for adequate caloric intake. She continued to fail to gain weight and underwent an EGD that demonstrated a duodenal ulcer. She was consequently started on sucralfate and omeprazole. Post-EGD lab work demonstrated a pronounced increase in AST and ALT. Pathology from the EGD biopsies later demonstrated viral inclusion bodies consistent with CMV duodenitis. Apart from malnutrition, other causes of immune deficiency were eliminated from the differential diagnosis due to negative HIV PCR and normal immunoglobulins. While on antiviral treatment, her viral load of 1080 IU/mL trended to resolution and her liver enzymes normalized. The patient was ultimately discharged home demonstrating adequate weight gain via gastrostomy tube feeds. This case advocates for pediatricians to include immunodeficiency and infectious etiologies in their differential for malnourished patients in order to lead to earlier diagnosis and management of this treatable condition.

## 1. Introduction

Cytomegalovirus (CMV) is a double stranded herpes virus and member of the Herpesviridae family. Other well studied viruses of this family include herpes simplex viruses (HSV) 1 and 2, Epstein-Barr virus, and varicella-zoster virus. CMV affects twenty to ninety percent of the general population [1]. It usually lies latent in immunocompetent hosts and therefore is often asymptomatic and undetected [2]. CMV, like HSV, can cause systemic diseases via reactivation in the setting of a weakened immune system. In immunocompromised patients such as patients with cancer, Human Immunodeficiency Virus (HIV), and organ transplantation, reactivation of CMV can increase morbidity and mortality [2]. CMV infection in immunocompromised patients often manifests with gastrointestinal complications. The colon is most often affected in the lower GI tract, while the esophagus and stomach are the sites most affected in the upper GI tract [3, 4]. CMV duodenitis, however, is a rare occurrence, especially in the pediatric population. Scant literature exists for such cases. Here, we present a case of a severely malnourished pediatric patient found to have CMV duodenitis.

## 2. Case Presentation

**Figure 1:**
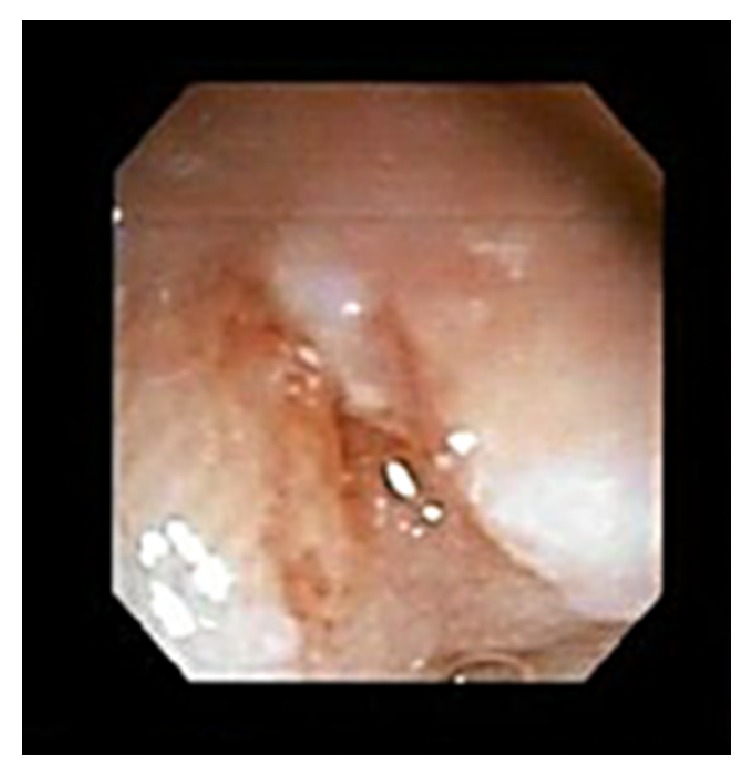
Duodenal ulcer in the duodenal bulb as seen on EGD.

**Figure 2:**
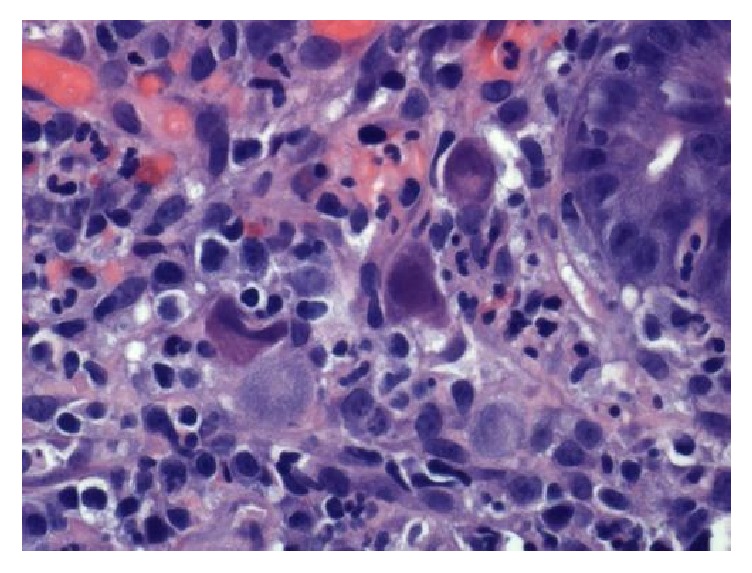
Duodenum biopsy showing CMV cytopathic effect.

**Figure 3:**
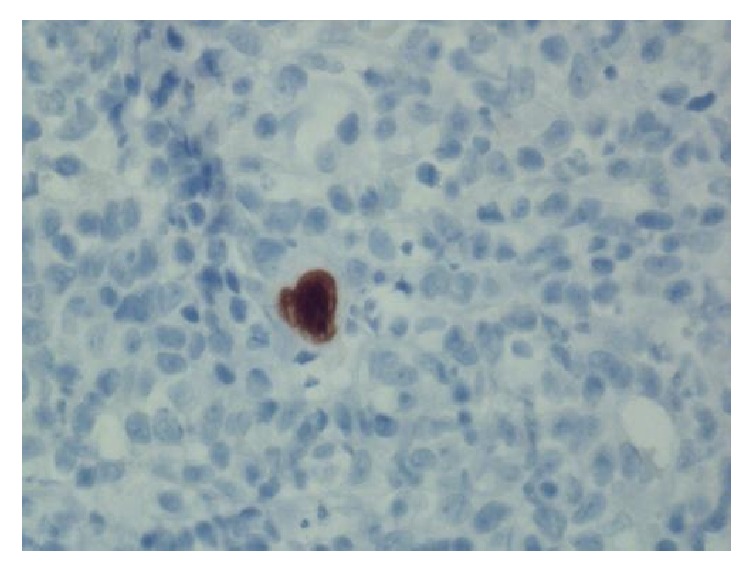
CMV immunostain.

A thirteen-month-old Vietnamese female with a history of atopic dermatitis presented to the Emergency Department with a seizure-like activity. She was found to be febrile at 102.2 degrees Fahrenheit with otherwise stable vital signs. Incidentally, her height and weight were concerning with a weight for age *z*-scores of −3.76. Review of systems was positive for a two-day history of rhinorrhea and dry cough. Review of systems was negative for previous fever, rash, and night sweats. Physical exam was remarkable for a severely underweight female with a normal neurological exam. Her abdominal exam was within normal limits with no appreciable hepatosplenomegaly. Skin exam showed mild eczematous changes on the flexural surfaces of bilateral upper and lower extremities. There was no jaundice. Initial workup included a complete blood count, blood culture, and urinalysis, all within normal limits. Her complete metabolic panel revealed a slightly elevated alanine aminotransferase (ALT) of 48 but normal aspartate aminotransferase (AST). She had no abnormal findings on repeat neurological exams. The patient was ultimately diagnosed with a simple febrile seizure likely secondary to a viral upper respiratory tract infection, but she was admitted for concerns for severe malnutrition. Upon admission, she was transitioned from her home diet consisting of only traditional Vietnamese soups to polymeric formula at 135 kcal/kg/day. Shortly after initiating formula feeds, her eczema worsened and she was therefore trialed on an elemental formula. Despite parental history of prior successful home oral feeds, hospital staff noted a significant oral aversion. Speech therapy evaluation ruled out motor and swallowing disorders. Although a trial with nasogastric feeds was initiated, she still demonstrated failure to gain weight. She consequently underwent an esophagogastroduodenoscopy (EGD), which demonstrated a duodenal ulcer in the duodenal bulb (Figure 1). At this time, she was started on omeprazole and sucralfate. Post-EGD lab work demonstrated elevated transaminases with ALT and AST at 112 and 340, respectively. A liver ultrasound revealed no abnormalities. Pathology from the EGD specimen biopsies later showed viral inclusion bodies consistent with CMV duodenitis (Figures 2 and 3).

**Figure 4:**
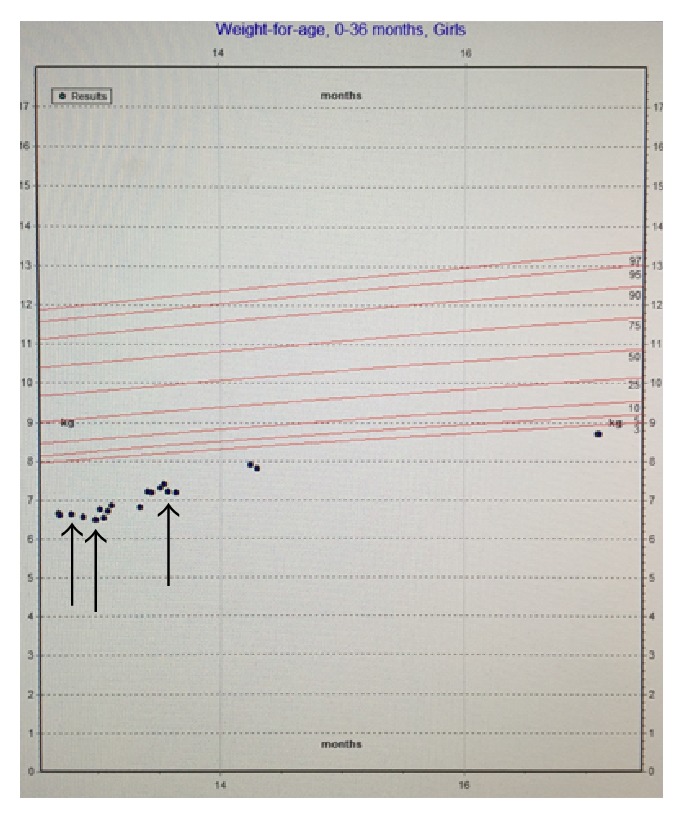
Patient's growth chart—weight for age. Arrows from left to right. (1) Initiation of nasogastric tube feeds. (2) Initiation of antiviral therapy. (3) Gastrostomy tube placement.

**Figure 5:**
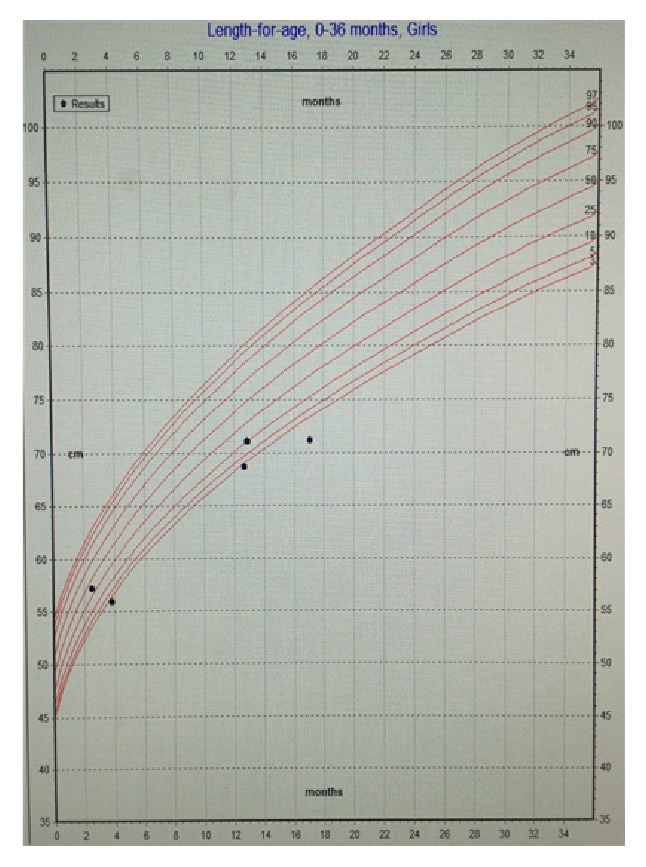
Patient's growth chart—length for age.

**Figure 6:**
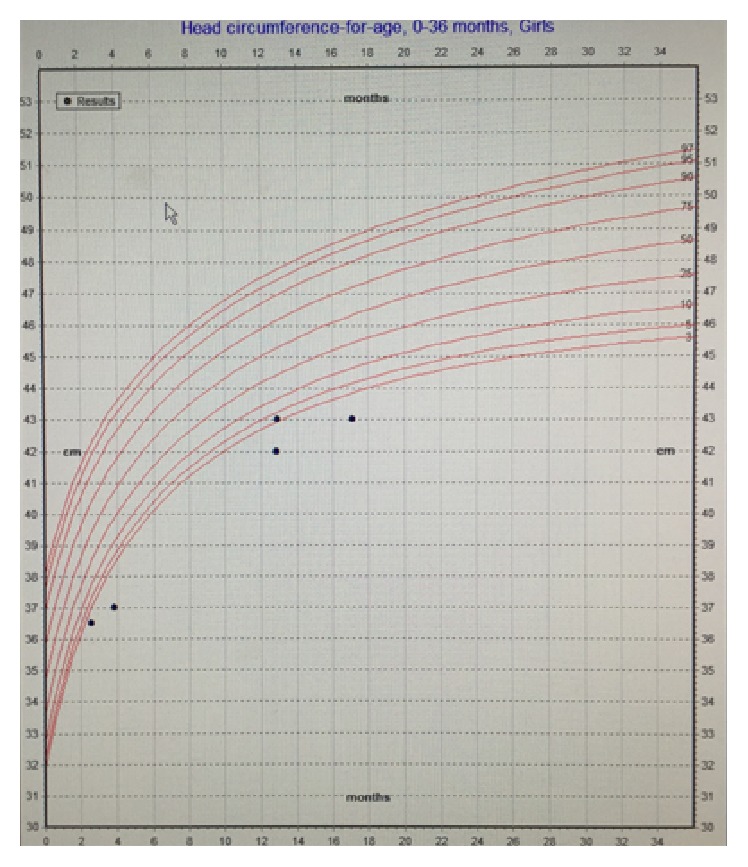
Patient's growth chart—head circumference for age.

Further workup in conjunction with ophthalmology ruled out CMV retinitis. Allergy and immunology were consulted in light of her progressive eczema and concern for compromised immunity. The patient had normal immunoglobulin (Ig) A and IgG levels, in addition to a negative HIV antigen/antibody screen. Her IgE levels were mildly elevated, most likely due to her atopy. Subsequently, she was started on topical emollient for her eczema. Further workup included normal plasma and urine amino acids and organic acids, ruling out major metabolic etiologies. Collaboration with the infectious diseases team found no additional viral infections such as hepatitis A, B, or C, parvovirus, adenovirus, or Epstein-Barr virus. The patient's CMV viral load at 1080 IU/mL was followed up via PCR and resolved with treatment consisting of intravenous ganciclovir followed by transition to oral valganciclovir. Her liver enzymes also normalized with treatment. Despite maximal pharmacologic therapy for her duodenal ulcer and CMV infection, she continued to refuse oral feeds. However, with nasogastric feeds, the patient demonstrated appropriate weight gain, and pediatric surgery proceeded with gastrostomy tube (g-tube) placement (Figures 4, 5, and 6). The patient was discharged home tolerating elemental formula via g-tube bolus feeds with daily weight gain. She was referred to outpatient feeding therapy upon discharge.

## 3. Discussion

CMV is a common herpes virus that affects all races, ages, and genders. The virus is typically classified by congenital or acquired transmission. CMV is one of the most common congenital viral infections with symptoms that include microcephaly, jaundice, hepatosplenomegaly, and rash. Infants with congenital CMV are at risk for developing hearing, vision, and developmental complications [5]. For pediatricians, any other presentation of CMV is uncommon.

It is well established that CMV is an opportunistic pathogen that can cause serious complications for people with weakened immune systems [2]. Older immunocompetent pediatric or adult patients infected with the virus are typically unaware of the diagnosis as they are usually asymptomatic. Symptomatic CMV in an immunocompetent older pediatric patient can present with malaise, fever, and sweats [6]. In our patient, the concerning signs included a fever and oral aversion in the setting of severe malnutrition. It is important for both general pediatricians and specialists alike to recognize the systemic and gastrointestinal symptoms indicative of CMV infection.

Though CMV can affect the entire body, CMV infection of the GI tract causes symptoms including pain, ulceration, bleeding, diarrhea, and perforation [7]. According to Bonetti et al., CMV of the upper GI tract most commonly affects the stomach, followed by the esophagus and the antrum [4]. In their review of thirty patients, duodenal involvement was rare and was only seen in one adult patient [3]. Our patient had an atypical location of her upper GI ulcer and associated CMV involvement. Nonetheless, CMV infection should be included in the differential diagnosis of gastrointestinal disease in immunocompromised patients.

Deficiencies in nutrients can impair growth and brain development and weaken the body's immune response to infection. In effect, malnutrition is the primary cause of immunodeficiency worldwide. The World Health Organization (WHO) previously recommended prophylactic antibiotic treatment for refeeding and severely malnourished patients with infectious symptoms [8]. In reviewing the patient's growth chart, she was severely malnourished before her febrile illness (Figure 4). The patient's severe malnutrition and resultant immune dysfunction placed her at risk for infection, despite negative workup for other common causes of immunodeficiency including HIV and immunoglobulin deficiency. Overall, her severe malnutrition was the cause of her immunodeficiency that eventually led to her CMV infection. This case demonstrates that pediatricians should have a heightened clinical suspicion for immunodeficiency and infectious symptoms in malnourished pediatric patients.

In our case presentation, both the conventional tissue biopsy and the serum PCR guided diagnosis and further case management. Specifically in our case, the improvement of symptoms with antiviral treatment paralleled the resolution of detected viral load via PCR (Figure 4). This congruency confirmed that the active CMV contributed to the oral aversion and subsequent malnutrition. Finally, it is imperative that pediatricians and team members understand that CMV disease is treatable.

## 4. Conclusion

Overall, this article describes a rare case and presentation of CMV infection of the duodenum in a pediatric patient. We consider the clinical presentation and course discussed in this case report to be useful for pediatricians due to the vast number of patients presenting with feeding difficulties and poor growth. This case advocates for pediatricians to include immunodeficiency and infectious etiology in their symptomatic and malnourished patients. We hope that this case report can perhaps lead to an earlier diagnosis and management of this treatable condition.
